# Brief reasons for living inventory: a psychometric investigation

**DOI:** 10.1186/s12888-017-1521-x

**Published:** 2017-11-06

**Authors:** Jan Christopher Cwik, Paula Siegmann, Ulrike Willutzki, Peter Nyhuis, Marcus Wolter, Thomas Forkmann, Heide Glaesmer, Tobias Teismann

**Affiliations:** 1 Mental Health Research and Treatment Center, Department of Psychology, Ruhr-Universität Bochum, Massenbergstrasse 11, 44787 Bochum, Germany; 2 Department for Psychology and Psychotherapy, Universität Witten/Herdecke, Witten, Germany; 3 St. Marien-Hospital Eickel, Herne, Germany; 4 Institute of Medical Psychology and Medical Sociology, University Hospital of RWTH Aachen University, Aachen, Germany; 5 Department of Medical Psychology and Medical Sociology, University Leipzig, Leipzig, Germany

**Keywords:** Suicide, Suicide ideation, Assessment, Reasons for living

## Abstract

**Background:**

The present study aimed at validating the German version of the Brief Reasons for Living inventory (BRFL).

**Methods:**

Validity and reliability were established in a community (*n* = 339) and a clinical sample (*n* = 272). Convergent and discriminant validity were investigated, and confirmatory factor analyses were conducted for the complete BRFL as well as for a 10-item version excluding conditional items on child-related concerns. Furthermore, it was assessed how BRFL scores moderate the association between depression and suicide ideation.

**Results:**

Results indicated an adequate fit of the data to the original factor structure. The total scale and the subscales of the German version of the BRFL had sufficient internal consistency, as well as good convergent and divergent validity. The BRFL demonstrated clinical utility by differentiating between participants with vs. without suicide ideation. Reasons for living proved to moderate the association between depression and suicide ideation.

**Conclusions:**

Results provide preliminary evidence that the BRFL may be a reliable and valid measure of adaptive reasons for living that can be used in clinic and research settings.

## Background

Worldwide, about 1 million people die by suicide each year, making it the 15th leading cause of death. It is estimated that for each adult who dies by suicide, there are likely to be more than 20 others with one or more suicide attempts [1] – as such suicide and suicide-related behaviors are a widely acknowledged major public health problem. While the majority of research in the field of suicidology has been directed at identifying risk factors for suicide ideation and behavior, far less attention has been given to adaptive, life-maintaining beliefs and expectations of persons contemplating suicide. However, the available literature consistently emphasizes that individuals who contemplate suicide often experience an “internal suicide debate” whereby they consider reasons for dying as well as reasons for living [2, 3]. Linehan and colleagues [4] introduced the Reasons for Living inventory to assess life-maintaining beliefs in non-suicidal persons and suicide ideators.

The Reasons for Living inventory [RFL; 4] is a 48-item self-report instrument, designed to evaluate a range of adaptive beliefs and expectations for living if suicide is considered. The inventory has six subscales: Survival and Coping Beliefs (e.g., “I believe I can find a purpose in life, a reason to live”), Responsibility to family (e.g., “My family depends on me and needs me”), Child-related concerns (“I want to watch my children as they grow”), Fear of Suicide (e.g., “I am afraid of death”), Fear of Social Disapproval (e.g., “I am concerned about what other people think of me”), and Moral Objections (e.g., “I consider it morally wrong”). The 48 items are scored on a 6-point scale ranging from (1) “not at all important” to (6) “extremely important”. Higher scores represent more reasons to live. The six-factor structure described by Linehan et al. [4] was replicated by Osman et al. [5–7]. Furthermore, the RFL showed acceptable internal consistency (Cronbachs α = .72–.92; see [4, 8]) as well as test-retest reliability (*r*_*tt*_ = .75–.85; see [8]). In clinical and nonclinical samples, the RFL has demonstrated convergent validity, correlating negatively with self-report measures of suicide ideation, suicide probability, depression and hopelessness [5, 9, 10]. In other investigations, the RFL has been shown to distinguish between (1) psychiatric inpatients and matched controls [11], (2) suicidal- and non-suicidal persons [5], as well as (3) depressed inpatients, who had attempted suicide and depressed inpatients, who had not attempted suicide [12]. Finally, a high score on the RFL predicted fewer suicide attempts within a 2-year period in women [13].

Taken together, the 48-item Reasons for Living inventory has demonstrated sound psychometric properties as well as clinical utility. Yet, its extensive length limits its utility in many institutional and screenings settings. Therefore, Ivanoff, Jang, Smyth, and Linehan [14] developed the 12-item Brief Reasons for Living inventory (BRFL). Using maximum-likelihood factor analysis, the original six-factor structure was replicated in a sample of 130 prison inmates. Two items represent each subscale. The BRFL total score and the RLF total score are highly correlated (*r* = .94) and the BRFL was shown to be a significant predictor of suicide ideation even after hopelessness and depression were simultaneously taken into account. Yet, the analysis was based on a limited number of prison inmates. Therefore, Ivanoff et al. [14] point to the necessity of cross-validating the BRFL in further clinical and non-clinical samples. Unlike the 48-item RFL, the BRFL has neither been studied extensively nor has it been translated to various languages. Therefore, the first aim of the current study was to validate a German version of the BRFL in a non-clinical and a clinical sample.

By doing so, we were also interested in finding out, whether reasons for living, as assessed by the BRFL, confer resilience, that is, buffer individuals against the development of suicide ideation and behavior. In their work on the buffering hypothesis, Johnson, Wood, Gooding, Taylor, and Tarrier [15] suggest that to be viewed as conferring resilience, a psychological construct needs to demonstrate the following characteristics: (1) It needs to comprise a separate dimension to risk and moderate the association between risk and outcome. Therefore, to ascertain resilience an assessment of both, risk and suicidality is necessary. (2) It needs to be viewed as existing on a bipolar continuum, with its inverse amplifying the association between risk and outcome. For example, high levels of social support or positive self-appraisals have been shown to have a buffering effect on suicidal thoughts, whereas low levels of social support or positive self-appraisals are an amplifying factor, increasing the association between risk and suicidality [16]. On this background, the second aim of the current study was to investigate whether reasons for living buffer the impact of depression – as a major risk factor for suicide [1] – on suicide ideation [9, 17].

## Methods

### Participants

Data was derived from two samples in Germany.

**Sample 1 (Online Sample)**: The first sample was a community sample of *N* = 339 participants of which *n* = 261 (77%) were female and *n* = 78 (23%) were male. Age ranged from 18 to 77 years, with a mean of 30.84 (*SD* = 12.82). Thirty-five participants (10.4%) had a suicide ideation score > 3 (measured with the Depressive Symptom Inventory – Suicidality Subscale, DSISS; [18]), which is the proposed cut-off score for serious suicide ideation. All participants were Caucasian. About 57% (*n* = 195) were students.

**Sample 2 (clinical sample)**: The second sample comprised *N* = 272 patients either being treated in a psychiatric inpatient unit (38.2%, *n* = 104) or an outpatient psychotherapeutic clinic (61.8%; *n* = 168). Of Sample 2, 170 participants (62.5%) were female and *n* = 102 (37.5%) were male. The mean of age was 40.43 years (*SD* = 12.86) ranging from 19 to 72. The most common diagnoses according to the International Classification of Diseases (ICD-[10, 19]) were affective disorders (51.1%), as well as neurotic, stress-related, and somatoform disorders (32.4%), followed by behavioral syndromes associated with physiological disturbances and physical factors (6.3%), personality disorders (4%), substance abuse (2.6%), psychotic disorders (1.1%) and other disorders (2.6%). Seventy-eight patients (29.3%) had a DSISS-score > 3 (with data on suicide ideation missing from seven patients). All participants were Caucasian.

Prior to assessments, the participants were informed about the purpose of the study, the voluntary nature of their participation, data storage and security. They gave written informed consent before participating. The study was approved by the responsible Ethics Committee.

### Procedure

**Sample 1 (online Sample)** was recruited between June 2016 and January 2017. Students at two universities in the Ruhr-region in Germany were approached and informed about receiving course credit for their participation. Furthermore, participants could share the link to the survey with others. The online survey was completed anonymously. The survey was programmed so that one could only proceed to the next questionnaire once all prior questions had been answered. Nonetheless, it was possible to quit the study at any time. All participants in Sample 1 were provided with information for receiving help in case of acute suicidality. Information about the national crisis hotline was given and also the offer to get in touch with the Centre for Psychotherapy of the Ruhr-Universität Bochum for support.

**Sample 2 (clinical sample)** was recruited between April 2016 and January 2017. Participants of this sample either underwent psychotherapy at a university outpatient clinic or at an inpatient psychiatric hospital in the Ruhr region in Germany. If inpatients had agreed to participate, questionnaires were presented in a paper-pencil version. No information on their identity was collected. The physician or therapist in charge of the patient’s treatment provided information on patient diagnoses. If outpatients had agreed to participate, they were asked to fill out the questionnaires online on a computer. All questions had to be answered in order to proceed to the next questionnaire. All participants of the clinical sample already received therapeutic help. Therefore, participants were informed to turn to the respective therapist in charge in case of suicidal thoughts or impulses.

### Measures

**Brief Reasons for Living inventory (BRFL)** [14]*.* The BRFL is a 12-item self-report measure intended to assess adaptive beliefs and expectations for living. The inventory has six subscales: fear of suicide, responsibility to family, survival and coping beliefs, child-related concerns, moral objections, and fear of social disapproval. The 12 items are scored on a 6-point scale ranging from (1) “not at all important” to (6) “extremely important”. Higher scores on the BRFL scale and on its subscales reflect more reasons for living. The German version of the BRFL was developed by means of a translation-back-translation procedure according to relevant guidelines for the translation of psychometric instruments [20].

**Depressive Symptom Inventory – Suicidality Subscale (DSI-SS)** [18]. The DSI-SS is a 4-item self-report questionnaire designed to assess the frequency and intensity of suicidal ideation and impulses in the past 2 weeks (“I am having thoughts about suicide and have formulated a definite plan”; “I always have thoughts of killing myself”; “In some situations I have impulses to kill myself”; “I am having thoughts about suicide and I am considering possible ways of doing it”). Scores on each item range from 0 to 3, with higher scores indicating greater severity of suicidal ideation. The first validation study of the German version of the DSI-SS [21] found good internal consistency (Cronbachs α = .90) for the scale. In accordance, internal consistency, as assessed with McDonald’s coefficient omega (ω) [22–24] was good in the online-sample, ω = .89, and in the clinical sample, ω = .94. This questionnaire was used to establish discriminant validity of BRFL, with the expectation that there would be significant negative associations between the BRFL and DSI-SS.

**Patient Health Questionnaire – Depression Module (PHQ-9)** [25]. Severity of depressive symptoms was measured by the PHQ-9. The PHQ-9 assesses the occurrence of nine depressive symptoms according to the Diagnostic and Statistical Manual of Mental Disorders [26] within the previous 2 weeks. It has been shown to have good sensitivity and specificity [27] as well as good internal consistency: Cronbach’s *α* ≥ .86 [28]. Internal consistency was ω = .86 in the online sample and ω = .85 in the clinical sample. This questionnaire was also used to establish discriminant validity of BRFL, with the expectation that there would be significant negative associations between the BRFL and PHQ-9. Additionally, this questionnaire was used to examine whether reasons for living moderates the association between depression and suicide ideation.

**Interpersonal Needs Questionnaire - Perceived Burdensomeness subscale (INQ-PB)** [29]. The INQ-PB assesses the amount of perceived burdensomeness with six items (e.g., “These days I feel like a burden on the people in my life”). All items are to be answered on a 7-point Likert scale ranging from “1” (not at all true for me) to “7” (very true for me). The German version of the INQ shows good psychometric properties [30]. Accordingly, internal consistency was good in the current study: ω = .89 in the online sample and ω = .94 in the clinical sample. The INQ-PB was also used to establish discriminant validity of BRFL, with the expectation that there would be significant negative associations with the BRFL.

**Positive Mental Health Scale (PMH)** [31]*.* The PMH-Scale assesses emotional, psychological and social aspects of well-being across 9 items (e.g., “I enjoy my life”), rated on a scale ranging from 0 (*do not agree*) to 3 (*agree*). The PMH is a person-centered questionnaire that consists of judgments across non-specific situations, thus constitutes a general measure of psychological functioning. Unidimensional structure and good convergent and discriminant validity are demonstrated in samples comprised of students, patients and the general population [31]. McDonald’s omega was ω = .92 in the online sample, and ω = .94 in the clinical sample. The PMH was used for establishing convergent validity of the BRFL, with the expectation that there would be significant positive associations between the scales.

**Social Support Scale (F-SozU)** [32]. Social support was assessed using the 14-item Social Support Scale measuring perceived and/or anticipated social support. Participants indicated agreement with statements such as “I experience a lot of understanding and security from others” on a 5-point Likert scale ranging from 1 (*not true)* to 5 (*true*). In a German sample, this unidimensional measure showed excellent Cronbach’s α and good convergent and discriminant validity [32]. Internal consistency in the current sample was ω = .94 in the online sample, and ω = .96 in the clinical sample. This questionnaire was also used to establish convergent validity of BRFL, with the expectation that there would be significant positive associations between the BRFL and F-SozU.

### Statistical analyses

In order to test the psychometric properties of the BRFL, an item analysis and a confirmatory factor analysis (CFA) were conducted. Considering the fact that only participants with children (*n* = 237) were able to answer Item 4 (“I want to watch my children as they grow”) and Item 7 (“The effect on my children could be harmful”), we decided to conduct all analyses for the six-factor model for those participants who had children, followed by analyses testing the five-factor model excluding the factor “child-related concerns” for the whole sample. To determine the fit of the six-factor and the five-factor solutions of the BRFL inventory, several goodness of fit indices were extracted for assessing the model fit: the relative χ2 (χ2/df), the root-mean-square-error-of-approximation (RMSEA) including the 90% confidence interval (90%-*CI*), the comparative-fit-index (CFI), the Tucker–Lewis index (TLI), and the standardized root-mean-square residual (SRMR). According to Hu and Bentler [33] and Hair et al. [34], cut-off values can be interpreted as follows: for the relative χ2, a value of <3 indicates a good model fit [35, 36]. RMSEA values of < .05 indicate a good model fit, whereas values between < .08 and > .05 can be seen as reasonable fit [37]. In case of the CFI and the TLI, values > .90 are indicators of an adequate fit, whereas values > .95 indicate a good fit [33, 38]. To represent a good model fit, SRMR values should be < .09 [34].

McDonald’s ω was calculated to determine the internal consistencies of the BRFL and subscales [22–24]. Results of the Kolmogorov-Smirnov-test [39, 40] illustrated that all items were not normally distributed, so that construct validity was tested via Spearman’s rank correlation analyses between the scales of the BRFL and the criterion measures. Group differences in BRFL scores between participants without suicide ideation (DSI-SS = 0) and participants with suicide ideation (DSI-SS ≥ 1) were tested with Mann-Whitney-U-Tests.

Finally, hierarchical regression analyses were conducted to examine whether reasons for living (BRFL-12 and BRFL-10) moderated the association between depression and suicide ideation. The variables were entered in four steps: In the first step of these analyses, group (clinical, online), age, and gender were entered as covariates. In the second step, depression severity – measured with the PHQ-9 excluding the suicidality item (Item 9) – was entered. In the third step, reasons for living – as measured with the BRFL-12 or BRFL-10 inventory – were included. In the final step, the interaction term of depression and reasons for living was entered. If the interaction term adds significant predictive variance to the regression model, it indicates a moderating effect of reasons for living on the association between depression and suicide ideation [41]. The magnitude of the interaction effect was assessed by the change in corrected *R* ^*2*^ (Δ*R* ^*2*^).

Diagnostics of multicollinearity revealed high multicollinearity (variance inflation factor (VIF) > 10 and/or tolerance coefficient < .2; see [33, 34, 42]) between PHQ-9 (VIF = 18.58, tolerance = .05) as well as the interaction between PHQ-9 and BRFL (VIF = 18.55, tolerance = .05) and other predictors. Thus, all continuous predictor variables were mean centered [43, 44], so that the lowest tolerance coefficient was .391, with a highest VIF of 2.556. Data analysis was conducted by using the statistic software program R [45], and its package lavaan (Version 0.5–23.1097) [46].

## Results

### Confirmatory factor analysis

**Fig. 1:**
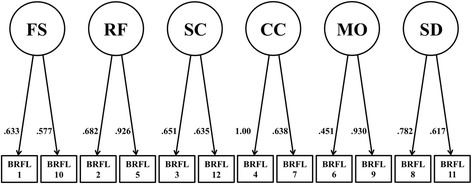
Structural equation model with pathways between the five factors of the Brief Reasons for Living inventory including child-related concerns (BRFL-12). BRFL-12 = Brief Reasons for Living Inventory including “child-related concerns” (*n* = 216); BRFL-FS = “fear of suicide” subscale; BRFL-RF = “responsibility to family” subscale; BRFL-SC = “survival and coping beliefs” subscale; BRFL-CC = “child-related concerns” subscale; BRFL-SD = “fear of social disapproval” subscale; BRFL-MO = “moral objections” subscale

**Fig. 2:**
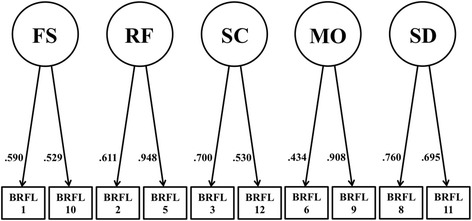
Structural equation model with pathways between the five factors of the Brief Reasons for Living inventory without child-related concerns (BRFL-10). BRFL-10 = Brief Reasons for Living Inventory without “child-related concerns” (*n* = 586); BRFL-FS = “fear of suicide” subscale; BRFL-RF = “responsibility to family” subscale; BRFL-SC = “survival and coping beliefs” subscale; BRFL-SD = “fear of social disapproval” subscale; BRFL-MO = “moral objections” subscale

Overall, the CFA revealed a very good model fit for the six-factor model (see Fig. 1) and for the five-factor model (see Fig. 2). For the six-factor model (*n* = 216), the CFA showed a relative χ^2^ of 1.53, which indicates a good model fit. Also the other fit indices indicated an adequate model fit: CFI = .965, TLI = .941, RMSEA = .050 (90%-*CI*: .021–.075), and SRMR = .043. Similarly, for the five-factor model (*n* = 586), the CFA showed a relative χ^2^ of 2.27, which still indicates a good model fit. Also for the five-factor model, the other fit indices indicated an adequate model: CFI = .964, TLI = .936, RMSEA = .050 (90%-*CI*: .032–.067), and SRMR = .032. All items showed medium to high standardized factor loadings on the assigned factors (see Fig. 1 and Fig. 2). Despite smaller sample sizes, the fit indices also indicated adequate model fit for the six-factor model, when the CFA was conducted separately for the online sample and the clinical sample. However, considering the very small sample sizes of participants with children in these groups, it was not possible to reveal reliable results for the five-factor model.

Standardized factor loadings for the six-factor model (BRFL-12) and the five-factor model (BRFL-10) are illustrated in Fig. 1 and Fig. 2. The majority of these factor loadings were moderate to high, ranging from .451 to 1.0 for the six-factor model and from .434 to .948 for the five-factor model. Both models showed comparable standardized factor loadings. However, the factor loadings for Item 6 were relatively low within both models.

### Scale properties

Internal consistency was assessed using Cronbach’s α. The internal consistency of the BRFL-12 (*n* = 216) was ω = .74; with ω = .75 in the clinical sample and ω = .73 in the online sample, whereas the internal consistency of the BRFL-10 (*n* = 586) was ω = .63; with ω = .73 in the clinical sample and ω = .52 in the online sample. Internal consistency for the subscales showed heterogeneous α coefficients between subscales and groups:

BRFL-FS: overall: ω = .48; clinical: ω = .54; online: ω = .42, BRFL-RF: overall: ω = .74; clinical: ω = .77; online: ω = .67, BRFL-SC: overall: ω = .55; clinical: ω = .57; online: ω = .49, BRFL-CC: overall: ω = .80; clinical: ω = .85; online: ω = .58, BRFL-MO: overall: ω = .56; clinical: ω = .52; online: ω = .58, BRFL-SD: overall: ω = .69; clinical: ω = .68; online: ω = .69. Taken item number into account, all Cronbach’s α coefficients were sufficient within both samples [47, 48]. Nonetheless, considering the small number of items of subscales, internal consistencies of subscales should be interpreted with caution.

The corrected item-total correlations for BRFL-FS ranged from *r*_*s*_ = .385 to .386, for BRFL-RF from *r*_*s*_ = .334 to .520, for BRFL-SC from *r*_*s*_ = .495 to .505, for BRFL-CC from *r*_*s*_ = .248 to .330, for BRFL-MO from *r*_*s*_ = .316 to .431, and for BRFL-SD from *r*_*s*_ = .343 to .394. Thus, the mean item-total correlations were in an optimal range [49]. With the exception of item 7 in BRFL-12 and item 6 in BRFL-10, all items showed acceptable corrected item-total correlations. Taken together, the retention of Item 6 and Item 7 is questionable, based on the low corrected item-total correlation (*r*_*s*_ < .30) [50].

Finally, except both items of the BRFL-CC subscale (Items: 4 and 7) – which at the most cannot be answered by most students – all items showed adequate item difficulty (see Table 1).

**Table 1:** Results of the item analysis of the Brief Reasons for Living Inventory (BRFL)

	BRFL-12	BRFL-10	
Item	Corrected item-total correlation	Corrected item-total correlation	Item difficulty
1. I am afraid of death. (BRFL-FS)	.386	.353	46.6
2. My family depends upon me and needs me. (BRFL-RF)	.334	.310	76.8
3. I do not want to die. (BRFL-SC)	.505	.394	69.6
4. The effect on my children could be harmful. (BRFL-CC)	.330	–	91.6
5. I love and enjoy my family too much and could net leave them. (BRFL-RF)	.520	.433	79.0
6. My religious beliefs forbid it. (BRFL-MO)	.316	.239	21.4
7. I want to watch my children as they grow. (BRFL-CC)	.248	–	88.2
8. I am concerned about what others would think of me. (BRFL-SD)	.394	.305	30.0
9. I consider it morally wrong. (BRFL-MO)	.431	.354	36.4
10. I am afraid of the actual “act” of killing myself (the pain, blood, violence). (BRFL-FS)	.385	.343	57.4
11. I would not want people to think I did not have control over my life. (BRFL-SD)	.343	.337	35.6
12. I believe I can find a purpose in life, a reason to live. (BRFL-SC)	.495	.359	76.6

*BRFL-12* Brief Reasons for Living Inventory including “child-related concerns” (*n* = 216), *BRFL-10* Brief Reasons for Living Inventory excluding “child-related concerns” (*n* = 586), *BRFL-FS* “fear of suicide” subscale, *BRFL-RF* “responsibility to family” subscale, *BRFL-SC* “survival and coping beliefs” subscale, *BRFL-CC* “child-related concerns” subscale, *BRFL-SD* “fear of social disapproval” subscale, *BRFL-MO* “moral objections” subscale

### Construct validity

Table 2 presents Spearman’s correlation coefficients between the BRFL and its subscales on the one hand, and the criterion measures on the other hand. Regarding the BRFL-12- and BRFL-10-total score, correlations with the F-SozU, the PMH-scale, the DSISS, the PHQ-9, and the INQ-PB were in the expected direction: More reasons for living were positively associated with more social support and positive mental health and negatively correlated with suicide ideation, depression and perceived burdensomeness. Different associations were found regarding the BRFL-subscales (see Table 2): Except for the BRFL-RF, BRFL-SC and the BRFL-FS subscales, only low and statistically non-significant associations were found regarding the other three BRFL-subscales.

**Table 2:** Results of Spearman’s rank correlation analyses between the Brief Reasons for Living Inventory and its subscales as well as questionnaires to measure construct validity

	BRFL-10	BRFL-FS	BRFL-RF	BRFL-SC	BRFL-CC	BRFL-MO	BRFL-SD	DSI-SS	F-SozU	PHQ-9	INQ-PB	PMH
BRFL-12	.969*** (*n* = 216)	.656*** (*n* = 216)	.583*** (*n* = 216)	.683*** (*n* = 216)	.270*** (*n* = 216)	.637*** (*n* = 216)	.601*** (*n* = 216)	−.444*** (*n* = 213)	.225** (*n* = 216)	−.173* (*n* = 211)	−.311*** (*n* = 213)	.204** (*n* = 215)
BRFL-10	–	.651*** (*n* = 586)	.569*** (*n* = 586)	.581*** (*n* = 586)	.070 (*n* = 216)	.576*** (*n* = 586)	.569*** (*n* = 586)	−.345*** (*n* = 581)	.278*** (*n* = 585)	−.176** (*n* = 580)	−.237*** (*n* = 580)	.227*** (*n* = 585)
BRFL-FS		–	.200*** (*n* = 602)	.330*** (*n* = 599)	.066 (*n* = 232)	.202*** (*n* = 601)	.202*** (*n* = 601)	−.172*** (*n* = 600)	.136** (*n* = 604)	−.044 (*n* = 596)	−.057 (*n* = 600)	.072 (*n* = 602)
BRFL-RF			–	.444*** (*n* = 597)	.330 (*n* = 230)	.155*** (*n* = 596)	.068 (*n* = 599)	−.383*** (*n* = 599)	.419*** (*n* = 602)	−.281*** (*n* = 595)	−.350*** (*n* = 599)	.341*** (*n* = 601)
BRFL-SC				–	.153* (*n* = 228)	.126** (*n* = 594)	.099* (*n* = 596)	−.426*** (*n* = 595)	.381*** (*n* = 599)	−.310*** (*n* = 594)	−.373*** (*n* = 595)	.354*** (*n* = 599)
BRFL-CC					–	.040 (*n* = 227)	.009 (*n* = 227)	.001 (*n* = 228)	−.062 (*n* = 232)	.042 (*n* = 226)	.080 (*n* = 229)	.015 (*n* = 230)
BRFL-MO						–	.323*** (*n* = 597)	−.104* (*n* = 594)	.018 (*n* = 599)	−.035 (*n* = 591)	−.065 (*n* = 594)	.084* (*n* = 597)
BRFL-SD							–	−.007 (*n* = 599)	−.070 (*n* = 601)	.068 (*n* = 595)	.062 (*n* = 598)	−.090* (*n* = 601)

*BRFL-12* Brief Reasons for Living Inventory, *BRFL-10* Brief Reasons for Living Inventory without child-related items, *BRFL-FS* “fear of suicide and death” subscale, *BRFL-RF* “responsibility to family”, *BRFL-SC* “survival and coping beliefs”, *BRFL-CC* “child-related concerns” subscale, *BRFL-MO* “moral objections” subscale, *BRFL-SD* “fear of social disapproval”, *DSI-SS* Depressive Symptom Inventory – Suicidality Subscale, *F-SozU* Social Support Scale, *PHQ-9* Patient Health Questionnaire (excluding item 9), *INQ-PB* Perceived Burdensomeness Subscale of the Interpersonal Needs Questionnaire, *PMH* Positive Mental Health Scale. * *p* < .05; ** *p* < .01; *** *p* < .001

Additionally, rank differences in all BRFL scores between participants without suicide ideation in the last 2 weeks (DSI-SS = 0) and participants with suicide ideation within the last 2 weeks (DSI-SS ≥ 1), were analyzed. Results illustrated generally higher ranks in the BRFL and all subscales in participants without suicide ideation compared to participants with suicide ideation. Yet, not all rank differences were statistically significant: The BRFL total score was significantly higher in participants without suicide ideation compared to participants with suicide ideation (BRFL-12: *U* = 3257.0, *p* < .001, *r* = .370; BRFL-10: *U* = 25,665.5, *p* < .001, *r* = .309). The same was true for four of the subscales: BRFL-FS: *U* = 23,190.5, *p* < .001, *r* = .156; BRFL-RF: *U* = 23,190.5, *p* < .001, *r* = .359; BRFL-SC: *U* = 23,190.5, *p* < .001, *r* = .383; BRFL-MO: *U* = 37,863.5, *p* = .015, *r* = .099. Yet, no significant differences were found on the other two subscales: BRFL-CC, *U* = 6549.5, *p* = .746, *r* = .021; BRFL-SD, *U* = 43,516.5, *p* = .990, *r* < .001.

Except the conditional child-related concerns factor, the remaining five factors/subscales were positively correlated with each other and the BRFL-sum scores (see Table 2). The mean total score of the BRFL-12 was 47.46 (*SD* = 10.08), ranging from 12 to 71 (clinical sample: *M* = 46.70; *SD* = 10.44; online sample: *M* = 48.97; *SD* = 9.21), whereas the mean total score of the BRFL-10 was 36.60 (*SD* = 7.90), ranging from 10 to 59 (clinical sample: *M* = 35.43; *SD* = 9.03; online sample: *M* = 37.46; *SD* = 6.85).

### Moderation analysis

Finally, in order to analyze whether reasons for living (BRFL-10) may moderate the association between depression (PHQ-9) and suicide ideation (DSI-SS), a multiple hierarchical regression analysis was conducted [51]. In the first step, group, age, and gender were entered as covariates. In the second step, PHQ-9 scores were entered. In the third step, BRFL-10-scores were entered. In the final step, the interaction between depressive symptoms and reasons for living (PHQ-9 x BRFL-10) was entered (see Table 3).

**Table 3:** Regression coefficients and model summaries of the six steps multiple hierarchical regression analysis including BRFL-10 (dependent variable: DSI-SS)

model	independent variable	β	95%-*CI*	*p*-value	*R* ^*2*^	Δ*R* ^*2*^
1	age	−.004	−.016–.008	.472	.079	.075
	group	−1.106	−1.434 - -.777	< .001		
	gender	.197	−.149–.543	.263		
2	age	−.003	−.015–.008	.597	.151	.145
	group	.215	−.273–.703	.387		
	gender	.351	.016–.686	.040		
	PHQ-9	.111	.080–.142	< .001		
3	age	−.002	−.013–.009	.767	.237	.230
	group	.193	−.270–.656	.414		
	gender	.243	−.076–.562	.136		
	PHQ-9	.095	.065–.125	< .001		
	BRFL-10	−.074	−.092 - -.056	< .001		
4	age	−.002	−.013–.009	.746	.250	.242
	group	.154	−.306–.614	.512		
	gender	.247	−.069–.564	.125		
	PHQ-9	.091	.061–.120	< .001		
	BRFL-10	−.067	−.086 - -.049	< .001		
	PHQ-9 x BRFL-10	−.004	−.006 - -.001	.002		

95%-CI = 95% confidence interval; Δ*R* ^*2*^ = corrected *R* ^*2*^; *PHQ-9* Patient Health Questionnaire (excluding item 9), *BRFL-10* Brief Reasons for Living inventory without child-related concerns

**Fig. 3:**
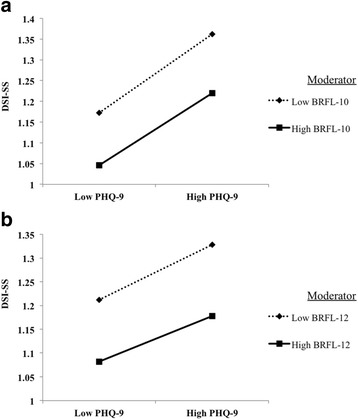
Moderation effect of (**a**) reasons for living inclusive children-related concerns and (**b**) reasons for living exclusive children-related concerns on the association between depression symptoms and suicidal thoughts and impulses (PHQ-9 responses were analyzed using a median split into low and high). DSI-SS = Depressive Symptom Inventory – Suicidality Subscale; PHQ-9 = Patient Health Questionnaire (excluding item 9); BRFL-12 = Brief Reasons for Living Inventory including “child-related concerns” (*n* = 216); BRFL-10 = Brief Reasons for Living Inventory excluding “child-related concerns”

As can be seen in Table 3, both reasons for living and depression were significantly associated with suicide ideation. Furthermore, there was a significant interaction between reasons for living and depression, indicating a moderation effect. Furthermore, model 4 presented a statistically significant Δ*R* ^2^ of .242, (*F*[6, 570] = 31.697; *p* < .001). Accordingly, the association between depression and suicide ideation was moderated by reasons for living (β = −.004; *t*[570] = −3.172; *p* = .002). Table 4 illustrates that analyzing the moderation of reasons for living with child-related concerns (BRFL-12) on the association between depression (PHQ-9) and suicide ideation (DSI-SS), revealed comparable results (Δ*R* ^2^ of .242, (*F*[6, 570] = 31.697; *p* < .001); β = −.004; *t*[570] = −3.172; *p* = .002). As shown in Fig. 3a-b, when participants’ responses in PHQ-9 were divided using a median split into low and high, for those participants who reported increased levels of reasons for living, lowered increases in the levels of suicide ideation at heightened severity of depression were found.

**Table 4:** Regression coefficients and model summaries of the six steps multiple hierarchical regression analysis including BRFL-12 (dependent variable: DSI-SS)

model	independent variable	β	95%-*CI*	*p*-value	*R* ^*2*^	Δ*R* ^*2*^
1	age	−.013	−.039–.013	.331	.087	.074
	group	−1.260	−1.895 - -.625	< .001		
	gender	.390	−.233–1.013	.219		
2	age	−.015	−.041–.010	.240	.124	.106
	group	−.189	−1.145 - .767	.697		
	gender	.637	.003–1.272	.049		
	PHQ-9	.090	.029–.151	.004		
3	age	−.009	−.033–.015	.465	.249	.230
	group	−.313	−1.201 - .576	.489		
	gender	.469	−.122–1.061	.119		
	PHQ-9	.063	.006–.120	.031		
	BRFL-12	−.082	−.110 - -.054	< .001		
4	age	−.009	−.032–.015	.473	.271	.250
	group	−.476	−1.363 - .411	.291		
	gender	.491	−.093–1.075	.099		
	PHQ-9	.053	−.004–.111	.066		
	BRFL-12	−.070	−.099 - -.041	< .001		
	PHQ-9 x BRFL-12	−.005	−.008 - -.001	.014		

95%-CI = 95% confidence interval; Δ*R* ^*2*^ = corrected *R* ^*2*^; *PHQ-9* Patient Health Questionnaire (excluding item 9), *BRFL-12* Brief Reasons for Living inventory

## Discussion

In the present study, the reliability and construct validity as well as the postulated factor structure of the German version of the Brief Reasons for Living Scale (BRFL) were investigated. Like the original version of the BRFL [14], the German version of the BRFL has sufficient internal consistency, as well as good convergent validity. The BRFL demonstrated initial clinical utility by differentiating among participants with vs. without suicide ideation: Across a non-clinical and a clinical sample, participants reporting suicide ideation also reported fewer reasons for living when considering suicide than did individuals without suicide ideation.

CFA revealed good model fit indices for both the six-factor (BRFL-12) and the five-factor model (BRFL-10). Accordingly, the assumed factor structure of the BRFL by Ivanoff et al. [14] can be confirmed in a German sample. Additionally, considering that the model of Ivanoff et al. [14] was based on prisoners, the results of the present study can also be seen as support for the assumed model in a more representative sample, including clinical patients. However, considering that subscales with only two items tend to be unstable [52, 53], more research regarding the stability of the model is still required. Taken item number into account, internal consistencies of the BRFL-12, BRFL-10 were sufficient. Nonetheless, internal consistency scores of some of the six subscales were rather low – especially in the online sample. This could be due to the fact that quite a few participants of the online sample had already experienced suicide ideation, making it rather difficult to answer questions dealing with suicide ideation.

Construct validity of the BRFL was supported by expected associations between the BRFL total scores and a set of relevant measures such as suicide ideation, depression, perceived burdensomeness, social support, and positive mental health. Particularly strong associations were found between the BRFL subscales “Responsibility to family”, “Survival and Coping Beliefs” and “Fear of suicide” with the different criterion measures. However, the BRFL subscales “Moral objections”, “Fear of social disapproval” and “Child-related Concerns” did not show substantial associations with the different criterion measures. One may speculate that – in a secular society such as Germany [54–56] – moral objections are only relevant to a small set of persons contemplating suicide. Low associations between the “Child-related Concerns” subscale and associated measures could be due to the fact that parents of small children may show a different response pattern to items such as “I want to watch my children as they grow”, than parents of older children [57]. Future studies on these aspects have to be awaited.

The current results complement previous research showing that reasons for living account for variance in suicide ideation that is not explained by other risk factors, such as depressive symptomatology [14]. As such, it was found that the BRFL significantly predicted suicide ideation – even after controlling for age, gender and depression. Furthermore, reasons for living emerged as a significant moderator of the depression-suicide ideation association. Those participants who reported a great number of reasons for living were less likely to experience suicide ideation even at the highest severity of depressive symptoms as compared to participants who reported low reasons for living. Reasons for living may therefore be considered as conferring resilience [15]. In line with this finding, suicide theorists and researchers have regularly argued that the suicidal state is characterized by ambivalence about life and death [58]. As such, Brown et al. [59] showed that the relative balance among the wish to live and the wish to die serves as a useful prospective risk factor for eventual death by suicide. In their study, psychiatric outpatients with a very strong orientation toward death were approximately 6.5 times more likely to die by suicide relative to those who were more ambivalent or oriented toward life.

Several limitations have to be considered when interpreting the current results. First, since 100% of the sample was Caucasian it is unclear how the findings would generalize to a more diverse population. However, considering that several studies showed significant construct variance between different countries (e.g., [60–62]), and the evidence of differences between individualistic and collectivistic cultures regarding reasons for suicide (e.g., [63, 64]), it could be possible that the reported results are not replicable in samples with other cultural backgrounds. Second, the conditional items of the “child-related concerns” factor are not applicable to individuals who do not have children and therefore constitute a considerable limitation of the scale. Thus, future studies should investigate the additional benefit of these items. Third, on one hand, even if the CFA showed adequate to good fit indices and a comparable structure was found for the adolescent version of the BRFL inventory [65], scales composing only two items can be methodologically problematic. On the other hand, as the examination of the construct validity of the BRFL inventory suggests, the subscales of the BRFL inventory are relatively indistinct. Furthermore, due to the inadequate internal consistency of many of the subscales and considering that the methods used for the extraction of factors in the original studies [4, 14] is somehow unclear, future studies investigating the six-factor model and alternative factor structures, the orthogonality of the factors as well as the applicability of a BRFL sum score are required. Fourth, as the present study investigated a clinical and an online sample, future studies with larger samples should address the issue of measurement invariance of the BRFL and its subscales. Fifth, the cross-sectional design of the current study precludes assertions about the temporal associations between study variables. Longitudinal studies on this issue are warranted. Sixth, the current study utilized only a self-report measure of suicide ideation. Future studies should also assess associations between reasons for living and suicidal behavior. In this regard, it would be of great interest to see whether reasons for living moderate the association between suicide ideation and suicide attempts.

Despite these limitations, the current results provide preliminary evidence that the BRFL may be a reliable and valid measure of adaptive reasons for living that could be used in clinic and research settings. Clinically, it can be used as an assessment as well as an intervention technique. As such, it might be used to identify protective themes that are most important to a person contemplating suicide. Clinicians might then focus on these themes with the intent of building for the future through the strengthening of life-oriented cognitions.

## Abbreviations

BRFL: Brief Reasons for Living inventory
BRFL-10: Brief reasons for living inventory 10-item version, excluding child-related concerns
BRFL-12: Brief reasons for living inventory 12-item version
BRFL-CC: Brief reasons for living inventory “child-related concerns” factor
BRFL-FS: Brief reasons for living inventory “fear of suicide” factor
BRFL-MO: Brief reasons for living inventory “moral objection” factor
BRFL-RF: Brief reasons for living inventory “responsibility to family” factor
BRFL-SC: Brief reasons for living inventory “survival and coping beliefs” factor
BRFL-SD: Brief reasons for living inventory “fear of social disapproval” factor
DSI-SS: Depressive symptom inventory – suicidality subscale
F-SozU: Social support scale
INQ-PB: Interpersonal needs questionnaire - perceived burdensomeness subscale
KMO: Kaiser-meyer-olkin measure of sampling adequacy
PCA: Principal component analysis
PHQ-9: Patient health questionnaire – depression module
PMH: Positive mental health scale
RFL: Reasons for living inventory
VIF: Variance inflation factor
